# A Parallel Biological Optimization Algorithm to Solve the Unbalanced Assignment Problem Based on DNA Molecular Computing

**DOI:** 10.3390/ijms161025338

**Published:** 2015-10-23

**Authors:** Zhaocai Wang, Jun Pu, Liling Cao, Jian Tan

**Affiliations:** 1 College of Information, Shanghai Ocean University, Shanghai 201306, China; E-Mail: zcwang1028@163.com; 2 Center for Finance and Accounting Research of University of International Business and Economics, Beijing 100029, China; E-Mail: junp1980@163.com; 3 College of Engineering Science and Technology, Shanghai Ocean University, Shanghai 201306, China; E-Mail: appll188@163.com; 4 Key Laboratory of Digital Earth Science, Institute of Remote Sensing and Digital Earth, Chinese Academy of Sciences, Beijing 100094, China

**Keywords:** DNA molecules computing, the unbalanced assignment problem, biological optimization algorithm, NP-complete problem

## Abstract

The unbalanced assignment problem (UAP) is to optimally resolve the problem of assigning *n* jobs to *m* individuals (*m* < *n*), such that minimum cost or maximum profit obtained. It is a vitally important Non-deterministic Polynomial (NP) complete problem in operation management and applied mathematics, having numerous real life applications. In this paper, we present a new parallel DNA algorithm for solving the unbalanced assignment problem using DNA molecular operations. We reasonably design flexible-length DNA strands representing different jobs and individuals, take appropriate steps, and get the solutions of the UAP in the proper length range and *O*(*mn*) time. We extend the application of DNA molecular operations and simultaneity to simplify the complexity of the computation.

## 1. Introduction

In the pathbreaking work of DNA computation, Adleman [1] firstly described how to solve a seven-node instance of a well-known NP-hard problem utilizing biological operations, and also demonstrated the potential parallel power of DNA computation. In 1995, Lipton [2] proved that Adleman’s experiment could be used to figure out the NP-complete satisfiability problem. DNA computation, as an interdisciplinary science using DNA molecular biotechnologies to solve conundrum problems of computer science and computational mathematics, has a wide application prospect in solving difficult problems. Huge storage capacity, massive parallelism and low energy consumption are primary advantages of DNA computation. The advantages imply that we can utilize DNA molecules to solve harder, larger problems such as NP-complete problems in linearly increasing time, in contrast to the exponentially increasing time required by an electronic computer. In recent years, DNA computation has received considerable interest from researchers. Some typical DNA computing models, such as the Adleman-Lipton model [1,2], the sticker model [3], the restriction enzyme model [4], the self-assembly model [5], the hairpin model [6], and the surface-based model [7], have already been established. Based on these models, lots of papers have been written for designing DNA procedures and algorithms to solve various NP-complete problems [8,9,10,11,12,13,14,15,16,17,18,19,20,21]. In order to fully understand the power of biological computation, it is worthwhile to try to solve more kinds of computationally-intractable problems with the aid of DNA biologic operations.

The assignment problem is a common topic in the fields related to operation management and network flow theory. This problem is known to be NP-hard and it is hard from a computational point of view as well. A standard assignment problem is to optimally resolve the problem of assigning *n* jobs to *m* individuals, such that minimum cost or maximum profit can be obtained. Various algorithms, including standard linear programming [6,7,8,9], Hungarian algorithm [10], neural network [11], and genetic algorithms [12], have been developed to find solutions. When we deal with real life situation, it becomes quite difficult to ensure that the number of jobs is exactly equal to individuals. Thus, the need arises to solve the unbalanced assignment problem in such a way that total assignment cost may be optimized along with the other constraints. In general, the unbalanced assignment problem can be considered as a particular case of the transportation problem, and can be formulated as a 0–1 integer linear programming [22,23]. The problem can be mathematically formulated as follows:

$$\text{min }Z=\sum_{i=1}^{m}\sum_{j=1}^{n}c_{ij}x_{ij}$$

$$s.t.\sum_{i=1}^{m}x_{ij}=1\;i=1,2,\cdots ,m$$

$$1\leq \sum_{j=1}^{n}x_{ij}\leq n-m+1\;j=1,2,\cdots ,n$$

$$x_{ij}=0,\text{ or }1$$

where the decision variable *x_ij_* = 1 means that the *j*-th job is assigned to the *i*-th individual; otherwise, *x_ij_* = 0 is in reverse; *c_ij_* is the associated cost incurred by the assignment (if *c_ij_* is the correlative profit, the object function will be max *Z*). For instance, the Table 1 defines the cost matrix A = [*c_ij_*]*_m_*_× *n*_ of the unbalanced assignment problem.

**Table 1:** Cost matrix A = [*c_ij_*]_3 × 5_.

Cost	*j*_1_	*j*_2_	*j*_3_	*j*_4_	*j*_5_
*i*_1_	5	9	1	2	7
*i*_2_	9	8	6	4	4
*i*_3_	4	7	8	5	2

In this paper, based on a combination of Adleman-Lipton model, A theoretically-efficient DNA algorithm is introduced for figuring out solutions of the unbalanced assignment problem, which is executed in *O*(*mn*) operations, where *n* is number of jobs and *m* is number of individuals. With the progress of molecular biology techniques, the proposed algorithm might be of practical use in treating medium-sized instances of UAP.

The rest of this paper is organized as follows. In Section 2, the Adleman-Lipton model is introduced in detail. Then, we use a DNA molecular algorithm for solving the unbalanced assignment problem. And prove DNA algorithm complexity and feasibility. In Section 3, we use computer to simulate the DNA experiment and get correct solution of the Table 1. We get conclusions in Section 4.

## 2. Results and Discussion

### 2.1. The Adleman-Lipton Model

The DNA operations proposed by Adleman [1] and Lipton [2] are described below. These operations will be used for figuring out solutions of the unbalanced assignment problem in this paper. In the Adleman-Lipton model: A (test) tube is a set of molecules of DNA (*i.e.*, a multi-set of finite strings over the alphabet {A,C,G,T}. Given a tube, one can perform the following operations:

(1) *Merge*(*T*_1_,*T*_2_): for two given test tubes *T*_1_ and *T*_2_, it stores the union *T*_1_∪*T*_2_ in *T*_1_ and leaves *T*_2_ empty;
(2) *Copy*(*T*_1_,*T*_2_): for a given test tube *T*_1_, it produces a test tube *T*_2_ with the same contents as *T*_1_;
(3) *Detect*(*T*): given a test tube *T*, it outputs “yes” if *T* contains at least one strand, otherwise, outputs “no”;
(4) *Separation*(*T*_1_,*X*,*T*_2_): for a given test tube *T*_1_ and a given set of strings *X*, it removes all single strands containing a string in *X* from *T*_1_, and produces a test tube *T*_2_ with the removed strands;
(5) *Selection*(*T*_1_,*L*,*T*_2_): for a given test tube *T*_1_ and a given integer *L*, it removes all strands with length *L* from *T*_1_, and produces a test tube *T*_2_ with the removed strands;
(6) *Sort*(*T*_1_,*T*_2_,*T*_3_): for a given test tube *T*_1_, it choose the shortest length strands in the tube *T*_2_, the longest strands in *T*_3_ and the remaining strands in *T*_1_;
(7) *Annealing*(*T*): for a given test tube *T*, it produces all feasible double strands in *T*. The produced double strands are still stored in *T* after annealing;
(8) *Denaturation*(*T*): for a given test tube *T*, it dissociates each double strand in *T* into two single strands;
(9) *Ligation*(*T*): for a given tube *T*, the operation is used to ligate together the strands in *T*;
(10) *Discard*(*T*): for a given test tube *T*, it discards the tube *T*;
(11) *Read*(*T*): for a given tube *T*, the operation is used to describe a single molecule, which is contained in the tube *T*. Even if *T* contains many different molecules each encoding a different set of bases, the operation can give an explicit description of exactly one of them;
(12) *Append-tail*(*T*,*Z*): for a given test tube *T* and a given DNA singled strand, it appends *Z* onto the end of every strand in the tube *T*.

Since these twelve manipulations are implemented with a constant number of biological steps for DNA strands [24,25], we assume that the complexity of each manipulation is in *O*(1) time steps.

### 2.2. DNA Algorithm for the Unbalanced Assignment Problem

#### 2.2.1. Thinking Process

The initial idea to solve the unbalanced assignment problem is as followed: generate strands corresponding to all possible job allocation schemes in a data pool, then, filter out inappropriate job allocation. Next, append the cost-weighted length strands in order to identify the schemes’ pros and cons. Finally, obtain the optimal solutions of the unbalanced assignment problem by using the corresponding DNA operations. Concretely, the proposed algorithm has four steps.

Step 1: Construct set *T* of all possible *m^n^* solutions for unbalanced assignment problem;
Step 2: For all possible solutions, eliminate inappropriate allocation, such as one job distribution to multiple individuals, one job without been assigned.
Step 3: Append time weight chain at the corresponding qualified strands in order to find the optimal solution.
Step 4: Get the shortest strands as the answer to the problem and identify the specific distribution.

#### 2.2.2. Detailed DNA Algorithm

Given a set of n jobs and m individuals (*m* < *n*), the unbalanced assignment problem requires that each job should be allocated to only one individual that the total cost, which is defined as the sum of the cost between each pair job and individual, is minimized. Consider a problem which consists of a set of *m* individuals *I* = {*i*_1_,*i*_2_,…,*i_m_*}. A set of n jobs *J* = {*j*_1_,*j*_2_,…,*j_n_*}is considered which are to be assigned for execution by *m* available individuals.

We suppose *m* < *n*. The execution cost of each job by all the individuals is known and mentioned in the matrix, namely A = [*c_ij_*]*_m_*_× *n*_ where *c_ij_* is the cost between job *j* and individual *i*. The objective is to determine the optimal assignment cost. A method is devised to obtain the said costs in such a way that all the jobs are to be allotted on the available individuals. In the following, the symbols *s*, *e*, *A_i_*, *B_j_* (*i* = 1,2,…,*m*, *j* = 1,2,…,*n*) denote distinct DNA single strands with the same length, say *t mer* (*t* is a positive integer, *mer* is monomer unit length). Obviously the length t of the DNA single strands greatly depends on the size of the problem involved in order to distinguish all above symbols [25]. Meanwhile we use the symbols *w_ij_* to denote the cost *c_ij_* and ||*w_ij_*|| = *c_ij_*. Then, in the below operations, we use the distinct DNA single strand symbols *sA_i_eB_j_* (*i* = 1,2,…,*m*, *j* = 1,2,…,*n*) to denote that the *j*-th job is assigned to the *i*-th individual without cost information. Simultaneity the symbols *s*, *e* are the signal of different edges division. Let

$$P=\{sA_{1}e,sA_{2}e,\cdots ,sA_{m}e\}$$

$$Q=\{\bar{eB_{1}s},\bar{eB_{2}s},\cdots ,\bar{eB_{n}s},B_{1},B_{2},\cdots ,B_{n}\}$$

For a cost matrix A = [*c_ij_*]*_m_*_× *n*_, the unbalanced assignment problem is firstly on the relation between *n* jobs and *m* individuals. Every possible assignment can be expressed by a list of DNA strands. DNA strands *sA_i_eB_j_* represent that the *j*-th job is assigned to the *i*-th individual. For example in Table 1, the DNA strands {sA_1_eB_2_sA_2_eB_3_sA_3_eB_4_sA_2_eB_1_sA_1_eB_5_} represent the 2 and 5-job to 1-individual, 1 and 3-job to 2-individual and 4-job to 3-individual. In this way, we can get DNA strands representing all possible *n* jobs to *m* individuals allocation relation.

(1) We choose all possible DNA strands denoting *n* jobs to *m* individuals allocation relation.

(1-1) *Merge*(*P*,*Q*);
(1-2) *Annealing*(*P*);
(1-3) *Ligation*(*P*);
(1-4) *Denaturation*(*P*);
(1-5) *Separation*(*P*,{*s*},*T*_1_);
(1-6) *Selection*(*T*_1_,4*nt*,*T*_2_).

This step operation can be finished in *O*(1) time steps since each manipulation above works in *O*(1).

(2) The unbalanced assignment problem requires that each job is assigned to a unique individual. So we check above DNA strands whether they satisfy the condition or not. The above assignment DNA strands should contain job strands *B_k_* (1 ≤ *k* ≤ *n*) one time only. For example in Table 1, the single strands {*sA*_1_*eB*_3_*sA*_3_*eB*_3_*sA*_2_*eB*_1_*sA*_1_*eB*_2_*sA*_2_*eB*_4_} ∈ *T*_2_ should be discarded for the 3-job simultaneously assigned to 1 and 3-individuals and not assigned the 5-job. We get all feasible assignment strands as follow:

For *k =* 1 to *k = n*
(2-1) *Separation*(*T*_2_,{*eB_k_s*},*T*_3_);
(2-2) *Discard*(*T*_2_);
(2-3) *Copy*(*T*_3_,*T*_2_);
(2-4) *Discard*(*T*_3_).
End for

In the above operations, we use one “For” clauses, thus this operation can be finished in *O*(*n*) time steps.

(3) In addition, the unbalanced assignment problem require that every individual get at least one job. So the above assignment DNA strands should contain individual strands *A_i_* (1 ≤ *i* ≤ *m*) at least one time. For example in Table 1, the single strands {*sA*_1_*eB*_3_*sA*_2_*eB*_4_*sA*_2_*eB*_1_*sA*_1_*eB*_2_*sA*_2_*eB*_5_} ∈ *T*_2_ should be discarded for not including the 3-individual. We get all feasible assignment strands as follow:

For *k =* 1 to *k = n*
(3-1) *Separation*(*T*_2_,{*sA_k_e*},*T*_4_);
(3-2) *Discard*(*T*_2_);
(3-3) *Copy*(*T*_4_,*T*_2_);
(3-4) *Discard*(*T*_4_).
End for

In the above operations, we use one “For” clause; thus, this operation can be finished in *O*(*m*) time steps.

(4) The solutions of unbalanced assignment problem must be with the minimum cost. In order to find the optimal results, we append the cost information strands at the end of above strands. For example, for the Table 1, the singled strands {*sA*_1_*eB*_3_*sA*_3_*eB*_2_*sA*_3_*eB*_4_*sA*_2_*eB*_1_*sA*_2_*eB*_3_} ∈ *T*_2_ representing the allocation: {*j*_3_→*i*_1_, *j*_2_→*i*_3_, *j*_4_→*i*_3_, *j*_1_→*i*_2_, *j*_5_→*i*_2_}, we append strands {*w*_14_, *w*_21_, *w*_32_, *w*_43_} at the above-mentioned strands to {*sA*_1_*eB*_3_*sA*_3_*eB*_2_*sA*_3_*eB*_4_*sA*_2_*eB*_1_*sA*_2_*eB*_3_*w*_13_*w*_32_*w*_34_*w*_21_*w*_25_}.

This is done by the following manipulations:

For *i* = 1 to *i* = *m*
   For *j* = 1 to *j* = *n*
(4-1) *Separation*(*T*_2_,{*sA_i_eB_j_*},*T*_5_);
(4-2) If (*Detect*(*T*_5_))
     Then execute (4-3) to (4-5)
(4-3) *Append-tail*(*T*_5_,*w_ij_*);
(4-4) *Merge*(*T*_2_,*T*_5_);
(4-5) *Discard*(*T*_5_).
   End for
End for

In the above operation, this operation can be finished in *O*(*mn*) time steps since we use two “For” clauses with *m* and *n* circulation.

(5) We take out those single strands in *T*_2_ with the shortest length, which give the solutions to the unbalanced assignment problem. For example in Table 1, those single strands in T_2_with shortest length are {*sA*_1_*eB*_3_*sA*_1_*eB*_4_*sA*_2_*eB*_2_*sA*_3_*eB*_1_*sA*_3_*eB*_5_*w*_13_*w*_14_*w*_22_*w*_31_*w*_35_}.

Therefore, solutions to unbalanced assignment problem for Table 1 are {*j*_3_→*i*_1_, *j*_4_→*i*_1_, *j*_2_→*i*_2_, *j*_1_→*i*_3_, *j*_5_→*i*_3_} with the weight sum 17.

(5-1) *Sort*(*T*_2_,*T*_5_,*T*_6_);
(5-2) *Read*(*T*_6_).

In the above operation, this operation can be finished in *O*(1) time steps since each single manipulation above works in *O*(1) steps. Finally the “Read” operation is applied to giving the exact solutions to the unbalanced assignment problem.

### 2.3. A Simple Example

We take a simple example in Table 2 to walk through the entire DNA algorithm.

**Table 2:** Cost matrix A = [*c_ij_*]_2 × 3_.

Cost	*j*_1_	*j*_2_	*j*_3_
*i*_1_	2	4	1
*i*_2_	1	2	3

After Step (1), all possible job assignment projects are shown in Table 3. For the unbalanced assignment problem, every job should be assigned to someone. The strands’ symbols after Step (2) are shown in Table 4. Table 5 displays the strand symbols, which mean every individual gets at least one job. Subsequently, the corresponding weight strands are attached to the original ones as shown in Table 6 and we get the solution strands in Table 7.

**Table 3:** **DNA Sequences symbols after Step (1) for Table 2.**

3′–5′ DNA Sequence	3′–5′ DNA Sequence	3′–5′ DNA Sequence	3′–5′ DNA Sequence
sA_1_eB_1_sA_1_eB_1_sA_1_eB_1_	sA_1_eB_1_sA_1_eB_1_sA_1_eB_2_	sA_1_eB_1_sA_1_eB_1_sA_1_eB_3_	sA_1_eB_1_sA_1_eB_1_sA_2_eB_1_
sA_1_eB_1_sA_1_eB_1_sA_2_eB_2_	sA_1_eB_1_sA_1_eB_1_sA_2_eB_3_	sA_1_eB_1_sA_1_eB_2_sA_1_eB_1_	sA_1_eB_1_sA_1_eB_2_sA_1_eB_2_
sA_1_eB_1_sA_1_eB_2_sA_1_eB_3_	sA_1_eB_1_sA_1_eB_2_sA_2_eB_1_	sA_1_eB_1_sA_1_eB_2_sA_2_eB_2_	sA_1_eB_1_sA_1_eB_2_sA_2_eB_3_
sA_1_eB_1_sA_1_eB_3_sA_1_eB_1_	sA_1_eB_1_sA_1_eB_3_sA_1_eB_2_	sA_1_eB_1_sA_1_eB_3_sA_1_eB_3_	sA_1_eB_1_sA_1_eB_3_sA_2_eB_1_
sA_1_eB_1_sA_1_eB_3_sA_2_eB_2_	sA_1_eB_1_sA_1_eB_3_sA_2_eB_3_	sA_1_eB_1_sA_2_eB_1_sA_1_eB_1_	sA_1_eB_1_sA_2_eB_1_sA_1_eB_2_
sA_1_eB_1_sA_2_eB_1_sA_1_eB_3_	sA_1_eB_1_sA_2_eB_1_sA_2_eB_1_	sA_1_eB_1_sA_2_eB_1_sA_2_eB_2_	sA_1_eB_1_sA_2_eB_1_sA_2_eB_3_
sA_1_eB_1_sA_2_eB_2_sA_1_eB_1_	sA_1_eB_1_sA_2_eB_2_sA_1_eB_2_	sA_1_eB_1_sA_2_eB_2_sA_1_eB_3_	sA_1_eB_1_sA_2_eB_2_sA_2_eB_1_
sA_1_eB_1_sA_2_eB_2_sA_2_eB_2_	sA_1_eB_1_sA_2_eB_2_sA_2_eB_3_	sA_1_eB_1_sA_2_eB_3_sA_1_eB_1_	sA_1_eB_1_sA_2_eB_3_sA_1_eB_2_
sA_1_eB_1_sA_2_eB_3_sA_1_eB_3_	sA_1_eB_1_sA_2_eB_3_sA_2_eB_1_	sA_1_eB_1_sA_2_eB_3_sA_2_eB_2_	sA_1_eB_1_sA_2_eB_3_sA_2_eB_3_
sA_1_eB_2_sA_1_eB_1_sA_1_eB_1_	sA_1_eB_2_sA_1_eB_1_sA_1_eB_2_	sA_1_eB_2_sA_1_eB_1_sA_1_eB_3_	sA_1_eB_2_sA_1_eB_1_sA_2_eB_1_
sA_1_eB_2_sA_1_eB_1_sA_2_eB_2_	sA_1_eB_2_sA_1_eB_1_sA_2_eB_3_	sA_1_eB_2_sA_1_eB_2_sA_1_eB_1_	sA_1_eB_2_sA_1_eB_2_sA_1_eB_2_
sA_1_eB_2_sA_1_eB_2_sA_1_eB_3_	sA_1_eB_2_sA_1_eB_2_sA_2_eB_1_	sA_1_eB_2_sA_1_eB_2_sA_2_eB_2_	sA_1_eB_2_sA_1_eB_2_sA_2_eB_3_
sA_1_eB_2_sA_1_eB_3_sA_1_eB_1_	sA_1_eB_2_sA_1_eB_3_sA_1_eB_2_	sA_1_eB_2_sA_1_eB_3_sA_1_eB_3_	sA_1_eB_2_sA_1_eB_3_sA_2_eB_1_
sA_1_eB_2_sA_1_eB_3_sA_2_eB_2_	sA_1_eB_2_sA_1_eB_3_sA_2_eB_3_	sA_1_eB_2_sA_2_eB_1_sA_1_eB_1_	sA_1_eB_2_sA_2_eB_1_sA_1_eB_2_
sA_1_eB_2_sA_2_eB_1_sA_1_eB_3_	sA_1_eB_2_sA_2_eB_1_sA_2_eB_1_	sA_1_eB_2_sA_2_eB_1_sA_2_eB_2_	sA_1_eB_2_sA_2_eB_1_sA_2_eB_3_
sA_1_eB_2_sA_2_eB_2_sA_1_eB_1_	sA_1_eB_2_sA_2_eB_2_sA_1_eB_2_	sA_1_eB_2_sA_2_eB_2_sA_1_eB_3_	sA_1_eB_2_sA_2_eB_2_sA_2_eB_1_
sA_1_eB_2_sA_2_eB_2_sA_2_eB_2_	sA_1_eB_2_sA_2_eB_2_sA_2_eB_3_	sA_1_eB_2_sA_2_eB_3_sA_1_eB_1_	sA_1_eB_2_sA_2_eB_3_sA_1_eB_2_
sA_1_eB_2_sA_2_eB_3_sA_1_eB_3_	sA_1_eB_2_sA_2_eB_3_sA_2_eB_1_	sA_1_eB_2_sA_2_eB_3_sA_2_eB_2_	sA_1_eB_2_sA_2_eB_3_sA_2_eB_3_
sA_1_eB_3_sA_1_eB_1_sA_1_eB_1_	sA_1_eB_3_sA_1_eB_1_sA_1_eB_2_	sA_1_eB_3_sA_1_eB_1_sA_1_eB_3_	sA_1_eB_3_sA_1_eB_1_sA_2_eB_1_
sA_1_eB_3_sA_1_eB_1_sA_2_eB_2_	sA_1_eB_3_sA_1_eB_1_sA_2_eB_3_	sA_1_eB_3_sA_1_eB_2_sA_1_eB_1_	sA_1_eB_3_sA_1_eB_2_sA_1_eB_2_
sA_1_eB_3_sA_1_eB_2_sA_1_eB_3_	sA_1_eB_3_sA_1_eB_2_sA_2_eB_1_	sA_1_eB_3_sA_1_eB_2_sA_2_eB_2_	sA_1_eB_3_sA_1_eB_2_sA_2_eB_3_
sA_1_eB_3_sA_1_eB_3_sA_1_eB_1_	sA_1_eB_3_sA_1_eB_3_sA_1_eB_2_	sA_1_eB_3_sA_1_eB_3_sA_1_eB_3_	sA_1_eB_3_sA_1_eB_3_sA_2_eB_1_
sA_1_eB_3_sA_1_eB_3_sA_2_eB_2_	sA_1_eB_3_sA_1_eB_3_sA_2_eB_3_	sA_1_eB_3_sA_2_eB_1_sA_1_eB_1_	sA_1_eB_3_sA_2_eB_1_sA_1_eB_2_
sA_1_eB_3_sA_2_eB_1_sA_1_eB_3_	sA_1_eB_3_sA_2_eB_1_sA_2_eB_1_	sA_1_eB_3_sA_2_eB_1_sA_2_eB_2_	sA_1_eB_3_sA_2_eB_1_sA_2_eB_3_
sA_1_eB_3_sA_2_eB_2_sA_1_eB_1_	sA_1_eB_3_sA_2_eB_2_sA_1_eB_2_	sA_1_eB_3_sA_2_eB_2_sA_1_eB_3_	sA_1_eB_3_sA_2_eB_2_sA_2_eB_1_
sA_1_eB_3_sA_2_eB_2_sA_2_eB_2_	sA_1_eB_3_sA_2_eB_2_sA_2_eB_3_	sA_1_eB_3_sA_2_eB_3_sA_1_eB_1_	sA_1_eB_3_sA_2_eB_3_sA_1_eB_2_
sA_1_eB_3_sA_2_eB_3_sA_1_eB_3_	sA_1_eB_3_sA_2_eB_3_sA_2_eB_1_	sA_1_eB_3_sA_2_eB_3_sA_2_eB_2_	sA_1_eB_3_sA_2_eB_3_sA_2_eB_3_
sA_2_eB_1_sA_1_eB_1_sA_1_eB_1_	sA_2_eB_1_sA_1_eB_1_sA_1_eB_2_	sA_2_eB_1_sA_1_eB_1_sA_1_eB_3_	sA_2_eB_1_sA_1_eB_1_sA_2_eB_1_
sA_2_eB_1_sA_1_eB_1_sA_2_eB_2_	sA_2_eB_1_sA_1_eB_1_sA_2_eB_3_	sA_2_eB_1_sA_1_eB_2_sA_1_eB_1_	sA_2_eB_1_sA_1_eB_2_sA_1_eB_2_
sA_2_eB_1_sA_1_eB_2_sA_1_eB_3_	sA_2_eB_1_sA_1_eB_2_sA_2_eB_1_	sA_2_eB_1_sA_1_eB_2_sA_2_eB_2_	sA_2_eB_1_sA_1_eB_2_sA_2_eB_3_
sA_2_eB_1_sA_1_eB_3_sA_1_eB_1_	sA_2_eB_1_sA_1_eB_3_sA_1_eB_2_	sA_2_eB_1_sA_1_eB_3_sA_1_eB_3_	sA_2_eB_1_sA_1_eB_3_sA_2_eB_1_
sA_2_eB_1_sA_1_eB_3_sA_2_eB_2_	sA_2_eB_1_sA_1_eB_3_sA_2_eB_3_	sA_2_eB_1_sA_2_eB_1_sA_1_eB_1_	sA_2_eB_1_sA_2_eB_1_sA_1_eB_2_
sA_2_eB_1_sA_2_eB_1_sA_1_eB_3_	sA_2_eB_1_sA_2_eB_1_sA_2_eB_1_	sA_2_eB_1_sA_2_eB_1_sA_2_eB_2_	sA_2_eB_1_sA_2_eB_1_sA_2_eB_3_
sA_2_eB_1_sA_2_eB_2_sA_1_eB_1_	sA_2_eB_1_sA_2_eB_2_sA_1_eB_2_	sA_2_eB_1_sA_2_eB_2_sA_1_eB_3_	sA_2_eB_1_sA_2_eB_2_sA_2_eB_1_
sA_2_eB_1_sA_2_eB_2_sA_2_eB_2_	sA_2_eB_1_sA_2_eB_2_sA_2_eB_3_	sA_2_eB_1_sA_2_eB_3_sA_1_eB_1_	sA_2_eB_1_sA_2_eB_3_sA_1_eB_2_
sA_2_eB_1_sA_2_eB_3_sA_1_eB_3_	sA_2_eB_1_sA_2_eB_3_sA_2_eB_1_	sA_2_eB_1_sA_2_eB_3_sA_2_eB_2_	sA_2_eB_1_sA_2_eB_3_sA_2_eB_3_
sA_2_eB_2_sA_1_eB_1_sA_1_eB_1_	sA_2_eB_2_sA_1_eB_1_sA_1_eB_2_	sA_2_eB_2_sA_1_eB_1_sA_1_eB_3_	sA_2_eB_2_sA_1_eB_1_sA_2_eB_1_
sA_2_eB_2_sA_1_eB_1_sA_2_eB_2_	sA_2_eB_2_sA_1_eB_1_sA_2_eB_3_	sA_2_eB_2_sA_1_eB_2_sA_1_eB_1_	sA_2_eB_2_sA_1_eB_2_sA_1_eB_2_
sA_2_eB_2_sA_1_eB_2_sA_1_eB_3_	sA_2_eB_2_sA_1_eB_2_sA_2_eB_1_	sA_2_eB_2_sA_1_eB_2_sA_2_eB_2_	sA_2_eB_2_sA_1_eB_2_sA_2_eB_3_
sA_2_eB_2_sA_1_eB_3_sA_1_eB_1_	sA_2_eB_2_sA_1_eB_3_sA_1_eB_2_	sA_2_eB_2_sA_1_eB_3_sA_1_eB_3_	sA_2_eB_2_sA_1_eB_3_sA_2_eB_1_
sA_2_eB_2_sA_1_eB_3_sA_2_eB_2_	sA_2_eB_2_sA_1_eB_3_sA_2_eB_3_	sA_2_eB_2_sA_2_eB_1_sA_1_eB_1_	sA_2_eB_2_sA_2_eB_1_sA_1_eB_2_
sA_2_eB_2_sA_2_eB_1_sA_1_eB_3_	sA_2_eB_2_sA_2_eB_1_sA_2_eB_1_	sA_2_eB_2_sA_2_eB_1_sA_2_eB_2_	sA_2_eB_2_sA_2_eB_1_sA_2_eB_3_
sA_2_eB_2_sA_2_eB_2_sA_1_eB_1_	sA_2_eB_2_sA_2_eB_2_sA_1_eB_2_	sA_2_eB_2_sA_2_eB_2_sA_1_eB_3_	sA_2_eB_2_sA_2_eB_2_sA_2_eB_1_
sA_2_eB_2_sA_2_eB_2_sA_2_eB_2_	sA_2_eB_2_sA_2_eB_2_sA_2_eB_3_	sA_2_eB_2_sA_2_eB_3_sA_1_eB_1_	sA_2_eB_2_sA_2_eB_3_sA_1_eB_2_
sA_2_eB_2_sA_2_eB_3_sA_1_eB_3_	sA_2_eB_2_sA_2_eB_3_sA_2_eB_1_	sA_2_eB_2_sA_2_eB_3_sA_2_eB_2_	sA_2_eB_2_sA_2_eB_3_sA_2_eB_3_
sA_2_eB_3_sA_1_eB_1_sA_1_eB_1_	sA_2_eB_3_sA_1_eB_1_sA_1_eB_2_	sA_2_eB_3_sA_1_eB_1_sA_1_eB_3_	sA_2_eB_3_sA_1_eB_1_sA_2_eB_1_
sA_2_eB_3_sA_1_eB_1_sA_2_eB_2_	sA_2_eB_3_sA_1_eB_1_sA_2_eB_3_	sA_2_eB_3_sA_1_eB_2_sA_1_eB_1_	sA_2_eB_3_sA_1_eB_2_sA_1_eB_2_
sA_2_eB_3_sA_1_eB_2_sA_1_eB_3_	sA_2_eB_3_sA_1_eB_2_sA_2_eB_1_	sA_2_eB_3_sA_1_eB_2_sA_2_eB_2_	sA_2_eB_3_sA_1_eB_2_sA_2_eB_3_
sA_2_eB_3_sA_1_eB_3_sA_1_eB_1_	sA_2_eB_3_sA_1_eB_3_sA_1_eB_2_	sA_2_eB_3_sA_1_eB_3_sA_1_eB_3_	sA_2_eB_3_sA_1_eB_3_sA_2_eB_1_
sA_2_eB_3_sA_1_eB_3_sA_2_eB_2_	sA_2_eB_3_sA_1_eB_3_sA_2_eB_3_	sA_2_eB_3_sA_2_eB_1_sA_1_eB_1_	sA_2_eB_3_sA_2_eB_1_sA_1_eB_2_
sA_2_eB_3_sA_2_eB_1_sA_1_eB_3_	sA_2_eB_3_sA_2_eB_1_sA_2_eB_1_	sA_2_eB_3_sA_2_eB_1_sA_2_eB_2_	sA_2_eB_3_sA_2_eB_1_sA_2_eB_3_
sA_2_eB_3_sA_2_eB_2_sA_1_eB_1_	sA_2_eB_3_sA_2_eB_2_sA_1_eB_2_	sA_2_eB_3_sA_2_eB_2_sA_1_eB_3_	sA_2_eB_3_sA_2_eB_2_sA_2_eB_1_
sA_2_eB_3_sA_2_eB_2_sA_2_eB_2_	sA_2_eB_3_sA_2_eB_2_sA_2_eB_3_	sA_2_eB_3_sA_2_eB_3_sA_1_eB_1_	sA_2_eB_3_sA_2_eB_3_sA_1_eB_2_
sA_2_eB_3_sA_2_eB_3_sA_1_eB_3_	sA_2_eB_3_sA_2_eB_3_sA_2_eB_1_	sA_2_eB_3_sA_2_eB_3_sA_2_eB_2_	sA_2_eB_3_sA_2_eB_3_sA_2_eB_3_

**Table 4:** DNA Sequences symbols after Step (2) for Table 2.

3′–5′ DNA Sequence	3′–5′ DNA Sequence	3′–5′ DNA Sequence	3′–5′ DNA Sequence
sA_1_eB_1_sA_1_eB_1_sA_2_eB_1_	sA_1_eB_1_sA_1_eB_1_sA_2_eB_2_	sA_1_eB_1_sA_1_eB_1_sA_2_eB_3_	sA_1_eB_1_sA_1_eB_2_sA_1_eB_1_
sA_1_eB_1_sA_1_eB_2_sA_1_eB_2_	sA_1_eB_1_sA_1_eB_2_sA_1_eB_3_	sA_1_eB_1_sA_1_eB_2_sA_2_eB_1_	sA_1_eB_1_sA_1_eB_2_sA_2_eB_2_
sA_1_eB_1_sA_1_eB_2_sA_2_eB_3_	sA_1_eB_1_sA_1_eB_3_sA_1_eB_1_	sA_1_eB_1_sA_1_eB_3_sA_1_eB_2_	sA_1_eB_1_sA_1_eB_3_sA_1_eB_3_
sA_1_eB_1_sA_1_eB_3_sA_2_eB_1_	sA_1_eB_1_sA_1_eB_3_sA_2_eB_2_	sA_1_eB_1_sA_1_eB_3_sA_2_eB_3_	sA_1_eB_1_sA_2_eB_1_sA_1_eB_1_
sA_1_eB_1_sA_2_eB_1_sA_1_eB_2_	sA_1_eB_1_sA_2_eB_1_sA_1_eB_3_	sA_1_eB_1_sA_2_eB_1_sA_2_eB_1_	sA_1_eB_1_sA_2_eB_1_sA_2_eB_2_
sA_1_eB_1_sA_2_eB_1_sA_2_eB_3_	sA_1_eB_1_sA_2_eB_2_sA_1_eB_1_	sA_1_eB_1_sA_2_eB_2_sA_1_eB_2_	sA_1_eB_1_sA_2_eB_2_sA_1_eB_3_
sA_1_eB_1_sA_2_eB_2_sA_2_eB_1_	sA_1_eB_1_sA_2_eB_2_sA_2_eB_2_	sA_1_eB_1_sA_2_eB_2_sA_2_eB_3_	sA_1_eB_1_sA_2_eB_3_sA_1_eB_1_
sA_1_eB_1_sA_2_eB_3_sA_1_eB_2_	sA_1_eB_1_sA_2_eB_3_sA_1_eB_3_	sA_1_eB_1_sA_2_eB_3_sA_2_eB_1_	sA_1_eB_1_sA_2_eB_3_sA_2_eB_2_
sA_1_eB_1_sA_2_eB_3_sA_2_eB_3_	sA_1_eB_2_sA_1_eB_1_sA_1_eB_1_	sA_1_eB_2_sA_1_eB_1_sA_1_eB_2_	sA_1_eB_2_sA_1_eB_1_sA_1_eB_3_
sA_1_eB_2_sA_1_eB_1_sA_2_eB_1_	sA_1_eB_2_sA_1_eB_1_sA_2_eB_2_	sA_1_eB_2_sA_1_eB_1_sA_2_eB_3_	sA_1_eB_2_sA_1_eB_2_sA_1_eB_1_
sA_1_eB_2_sA_1_eB_2_sA_1_eB_2_	sA_1_eB_2_sA_1_eB_2_sA_1_eB_3_	sA_1_eB_2_sA_1_eB_2_sA_2_eB_1_	sA_1_eB_2_sA_1_eB_2_sA_2_eB_2_
sA_1_eB_2_sA_1_eB_2_sA_2_eB_3_	sA_1_eB_2_sA_1_eB_3_sA_1_eB_1_	sA_1_eB_2_sA_1_eB_3_sA_1_eB_2_	sA_1_eB_2_sA_1_eB_3_sA_1_eB_3_
sA_1_eB_2_sA_1_eB_3_sA_2_eB_1_	sA_1_eB_2_sA_1_eB_3_sA_2_eB_2_	sA_1_eB_2_sA_1_eB_3_sA_2_eB_3_	sA_1_eB_2_sA_2_eB_1_sA_1_eB_1_
sA_1_eB_2_sA_2_eB_1_sA_1_eB_2_	sA_1_eB_2_sA_2_eB_1_sA_1_eB_3_	sA_1_eB_2_sA_2_eB_1_sA_2_eB_1_	sA_1_eB_2_sA_2_eB_1_sA_2_eB_2_
sA_1_eB_2_sA_2_eB_1_sA_2_eB_3_	sA_1_eB_2_sA_2_eB_2_sA_1_eB_1_	sA_1_eB_2_sA_2_eB_2_sA_1_eB_2_	sA_1_eB_2_sA_2_eB_2_sA_1_eB_3_
sA_1_eB_2_sA_2_eB_2_sA_2_eB_1_	sA_1_eB_2_sA_2_eB_2_sA_2_eB_2_	sA_1_eB_2_sA_2_eB_2_sA_2_eB_3_	sA_1_eB_2_sA_2_eB_3_sA_1_eB_1_
sA_1_eB_2_sA_2_eB_3_sA_1_eB_2_	sA_1_eB_2_sA_2_eB_3_sA_1_eB_3_	sA_1_eB_2_sA_2_eB_3_sA_2_eB_1_	sA_1_eB_2_sA_2_eB_3_sA_2_eB_2_
sA_1_eB_2_sA_2_eB_3_sA_2_eB_3_	sA_1_eB_3_sA_1_eB_1_sA_1_eB_1_	sA_1_eB_3_sA_1_eB_1_sA_1_eB_2_	sA_1_eB_3_sA_1_eB_1_sA_1_eB_3_
sA_1_eB_3_sA_1_eB_1_sA_2_eB_1_	sA_1_eB_3_sA_1_eB_1_sA_2_eB_2_	sA_1_eB_3_sA_1_eB_1_sA_2_eB_3_	sA_1_eB_3_sA_1_eB_2_sA_1_eB_1_
sA_1_eB_3_sA_1_eB_2_sA_1_eB_2_	sA_1_eB_3_sA_1_eB_2_sA_1_eB_3_	sA_1_eB_3_sA_1_eB_2_sA_2_eB_1_	sA_1_eB_3_sA_1_eB_2_sA_2_eB_2_
sA_1_eB_3_sA_1_eB_2_sA_2_eB_3_	sA_1_eB_3_sA_1_eB_3_sA_1_eB_1_	sA_1_eB_3_sA_1_eB_3_sA_1_eB_2_	sA_1_eB_3_sA_1_eB_3_sA_1_eB_3_
sA_1_eB_3_sA_1_eB_3_sA_2_eB_1_	sA_1_eB_3_sA_1_eB_3_sA_2_eB_2_	sA_1_eB_3_sA_1_eB_3_sA_2_eB_3_	sA_1_eB_3_sA_2_eB_1_sA_1_eB_1_
sA_1_eB_3_sA_2_eB_1_sA_1_eB_2_	sA_1_eB_3_sA_2_eB_1_sA_1_eB_3_	sA_1_eB_3_sA_2_eB_1_sA_2_eB_1_	sA_1_eB_3_sA_2_eB_1_sA_2_eB_2_
sA_1_eB_3_sA_2_eB_1_sA_2_eB_3_	sA_1_eB_3_sA_2_eB_2_sA_1_eB_1_	sA_1_eB_3_sA_2_eB_2_sA_1_eB_2_	sA_1_eB_3_sA_2_eB_2_sA_1_eB_3_
sA_1_eB_3_sA_2_eB_2_sA_2_eB_1_	sA_1_eB_3_sA_2_eB_2_sA_2_eB_2_	sA_1_eB_3_sA_2_eB_2_sA_2_eB_3_	sA_1_eB_3_sA_2_eB_3_sA_1_eB_1_
sA_1_eB_3_sA_2_eB_3_sA_1_eB_2_	sA_1_eB_3_sA_2_eB_3_sA_1_eB_3_	sA_1_eB_3_sA_2_eB_3_sA_2_eB_1_	sA_1_eB_3_sA_2_eB_3_sA_2_eB_2_
sA_1_eB_3_sA_2_eB_3_sA_2_eB_3_	sA_2_eB_1_sA_1_eB_1_sA_1_eB_1_	sA_2_eB_1_sA_1_eB_1_sA_1_eB_2_	sA_2_eB_1_sA_1_eB_1_sA_1_eB_3_

**Table 5:** DNA Sequences symbols after Step (1) for Table 2.

3′–5′ DNA Sequence	3′–5′ DNA Sequence	3′–5′ DNA Sequence	3′–5′ DNA Sequence
sA_1_eB_1_sA_1_eB_2_sA_2_eB_3_	sA_1_eB_1_sA_1_eB_3_sA_2_eB_2_	sA_1_eB_1_sA_2_eB_2_sA_1_eB_3_	sA_1_eB_1_sA_2_eB_2_sA_2_eB_3_
sA_1_eB_1_sA_2_eB_3_sA_1_eB_2_	sA_1_eB_1_sA_2_eB_3_sA_2_eB_2_	sA_1_eB_2_sA_1_eB_1_sA_2_eB_3_	sA_1_eB_2_sA_1_eB_3_sA_2_eB_1_
sA_1_eB_2_sA_2_eB_1_sA_1_eB_3_	sA_1_eB_2_sA_2_eB_1_sA_2_eB_3_	sA_1_eB_2_sA_2_eB_3_sA_1_eB_1_	sA_1_eB_2_sA_2_eB_3_sA_2_eB_1_
sA_1_eB_3_sA_1_eB_1_sA_2_eB_2_	sA_1_eB_3_sA_1_eB_2_sA_2_eB_1_	sA_1_eB_3_sA_2_eB_1_sA_1_eB_2_	sA_1_eB_3_sA_2_eB_1_sA_2_eB_2_
sA_1_eB_3_sA_2_eB_2_sA_1_eB_1_	sA_1_eB_3_sA_2_eB_2_sA_2_eB_1_	sA_2_eB_1_sA_1_eB_2_sA_1_eB_3_	sA_2_eB_1_sA_1_eB_2_sA_2_eB_3_
sA_2_eB_1_sA_1_eB_3_sA_1_eB_2_	sA_2_eB_1_sA_1_eB_3_sA_2_eB_2_	sA_2_eB_1_sA_2_eB_2_sA_1_eB_3_	sA_2_eB_1_sA_2_eB_3_sA_1_eB_2_
sA_2_eB_2_sA_1_eB_1_sA_1_eB_3_	sA_2_eB_2_sA_1_eB_1_sA_2_eB_3_	sA_2_eB_2_sA_1_eB_3_sA_1_eB_1_	sA_2_eB_2_sA_1_eB_3_sA_2_eB_1_
sA_2_eB_2_sA_2_eB_1_sA_1_eB_3_	sA_2_eB_2_sA_2_eB_3_sA_1_eB_1_	sA_2_eB_3_sA_1_eB_1_sA_1_eB_2_	sA_2_eB_3_sA_1_eB_1_sA_2_eB_2_
sA_2_eB_3_sA_1_eB_2_sA_1_eB_1_	sA_2_eB_3_sA_1_eB_2_sA_2_eB_1_	sA_2_eB_3_sA_2_eB_1_sA_1_eB_2_	sA_2_eB_3_sA_2_eB_2_sA_1_eB_1_

**Table 6:** DNA Sequences symbols after Step (4) for Table 2.

3′–5′ DNA Sequence	3′–5′ DNA Sequence	3′–5′ DNA Sequence
sA_1_eB_1_sA_1_eB_2_sA_2_eB_3_w_11_w_12_w_23_	sA_1_eB_1_sA_1_eB_3_sA_2_eB_2_w_11_w_13_w_22_	sA_1_eB_1_sA_2_eB_2_sA_1_eB_3_w_11_w_13_w_22_
sA_1_eB_1_sA_2_eB_2_sA_2_eB_3_w_11_w_22_w_23_	sA_1_eB_1_sA_2_eB_3_sA_1_eB_2_w_11_w_12_w_23_	sA_1_eB_1_sA_2_eB_3_sA_2_eB_2_w_11_w_22_w_23_
sA_1_eB_2_sA_1_eB_1_sA_2_eB_3_w_11_w_12_w_23_	sA_1_eB_2_sA_1_eB_3_sA_2_eB_1_w_12_w_13_w_21_	sA_1_eB_2_sA_2_eB_1_sA_1_eB_3_w_12_w_13_w_21_
sA_1_eB_2_sA_2_eB_1_sA_2_eB_3_w_12_w_21_w_23_	sA_1_eB_2_sA_2_eB_3_sA_1_eB_1_w_11_w_12_w_23_	sA_1_eB_2_sA_2_eB_3_sA_2_eB_1_w_12_w_21_w_23_
sA_1_eB_3_sA_1_eB_1_sA_2_eB_2_w_11_w_13_w_22_	sA_1_eB_3_sA_1_eB_2_sA_2_eB_1_w_12_w_13_w_21_	sA_1_eB_3_sA_2_eB_1_sA_1_eB_2_w_12_w_13_w_21_
sA_1_eB_3_sA_2_eB_1_sA_2_eB_2_w_13_w_21_w_22_	sA_1_eB_3_sA_2_eB_2_sA_1_eB_1_w_11_w_13_w_22_	sA_1_eB_3_sA_2_eB_2_sA_2_eB_1_w_13_w_21_w_22_
sA_2_eB_1_sA_1_eB_2_sA_1_eB_3_w_12_w_13_w_21_	sA_2_eB_1_sA_1_eB_2_sA_2_eB_3_w_12_w_21_w_23_	sA_2_eB_1_sA_1_eB_3_sA_1_eB_2_w_12_w_13_w_21_
sA_2_eB_1_sA_1_eB_3_sA_2_eB_2_w_13_w_21_w_22_	sA_2_eB_1_sA_2_eB_2_sA_1_eB_3_w_13_w_21_w_22_	sA_2_eB_1_sA_2_eB_3_sA_1_eB_2_w_12_w_21_w_23_
sA_2_eB_2_sA_1_eB_1_sA_1_eB_3_w_11_w_13_w_22_	sA_2_eB_2_sA_1_eB_1_sA_2_eB_3_w_11_w_22_w_23_	sA_2_eB_2_sA_1_eB_3_sA_1_eB_1_w_11_w_13_w_22_
sA_2_eB_2_sA_1_eB_3_sA_2_eB_1_w_13_w_21_w_22_	sA_2_eB_2_sA_2_eB_1_sA_1_eB_3_w_13_w_21_w_22_	sA_2_eB_2_sA_2_eB_3_sA_1_eB_1_w_11_w_22_w_23_
sA_2_eB_3_sA_1_eB_1_sA_1_eB_2_w_11_w_12_w_23_	sA_2_eB_3_sA_1_eB_1_sA_2_eB_2_w_11_w_22_w_23_	sA_2_eB_3_sA_1_eB_2_sA_1_eB_1_w_11_w_12_w_23_
sA_2_eB_3_sA_1_eB_2_sA_2_eB_1_w_12_w_21_w_23_	sA_2_eB_3_sA_2_eB_1_sA_1_eB_2_w_12_w_21_w_23_	sA_2_eB_3_sA_2_eB_2_sA_1_eB_1_w_11_w_22_w_23_

**Table 7:** DNA Sequences symbols after Step (5) for Table 2.

3′–5′ DNA Sequence	3′–5′ DNA Sequence	3′–5′ DNA Sequence
sA_1_eB_3_sA_2_eB_1_sA_2_eB_2_w_13_w_21_w_22_	sA_1_eB_3_sA_2_eB_2_sA_2_eB_1_w_13_w_21_w_22_	sA_2_eB_1_sA_1_eB_3_sA_2_eB_2_w_13_w_21_w_22_
sA_2_eB_1_sA_2_eB_2_sA_1_eB_3_w_13_w_21_w_22_	sA_2_eB_2_sA_1_eB_3_sA_2_eB_1_w_13_w_21_w_22_	sA_2_eB_2_sA_2_eB_1_sA_1_eB_3_w_13_w_21_w_22_

### 2.4. The Complexity and Feasibility of the Proposed DNA Algorithm

The following theorem tells that the algorithm proposed above really can get solutions of the unbalanced assignment problem in *O*(*mn*) steps using DNA molecules.

**Theorem 1.** *The solutions of the unbalanced assignment problem with n jobs and m individuals can be obtained by the above DNA operations.*

**Proof.** We first get all combinations of the *n* jobs assignment in the data pool after the first step. Since the unbalanced assignment problem requires every job be assigned, we discard DNA strands without some jobs information at step (2). Additionally, each individual should get at least one job in the problem, thus we abandon DNA strands without some jobs information at step (3). Simultaneity is required in order to find the minimum solution, thus we append the corresponding cost strands at the end of previous strands at step (4). The shortest stands in the tube *T*_6_ mean the solutions to the unbalanced assignment problem, and we can “read” the answer at the last step.

**Theorem 2.** *The solutions of the unbalanced assignment problem with n jobs and m individuals can be figured out in O(mn) time steps using DNA molecules computation.*

**Proof.** We get that the complexity of every biological operation is in *O*(1) time [14], the manipulation of the total algorithm can be entirely finished in a finite time range, such as step (1), (5) in *O*(1) time, step (2) in *O*(*n*), step (3) in *O*(*m*), and step (4) in *O*(*mn*). The time complexity *T* of the total algorithm is as follows:

*T*(Step (1)) = *O*(1);
*T*(Step (2)) = *O*(*n*);
*T*(Step (3)) = *O*(*m*);
*T*(Step (4)) = *O*(*mn*);
*T*(Step (4)) = *O*(1);
*T* = *T*(Step (1)) + *T*(Step (2)) + *T*(Step (3)) + *T*(Step (4)) + *T*(Step (5));
= *O*(1) + *O*(*n*) + *O*(*m*) + *O*(*mn*) + *O*(1);
= *O*(*mn*).

**Theorem 3.** *The solutions strands of the unbalanced assignment problem with n jobs and m individuals can be found in the finite length range.*

**Proof.** After the second step, we pick up the DNA strands for all possible assignment choices with *n* jobs and *m* individuals. Here the single strands in tube *T*_2_ at step (3) can be described:

$$sA_{i_{1}}eB_{j_{1}}sA_{i_{2}}eB_{j_{2}}\cdots sA_{i_{p}}eB_{j_{p}}\cdots sA_{i_{n}}eB_{j_{n}}\;i_{p}\in \{1,2,\cdots ,m\},j_{p}\in \{1,2,\cdots ,n\}$$

In the beginning we reasonably design the length of *s*, *e*, *A_k_*, *B_k_*, for

$$\left\|s\right\|=\left\|A_{k}\right\|=\left\|B_{k}\right\|=\left\|e\right\|=t\;mer$$

In order to choose the minimum cost assignment, we append the cost strands *w_ij_* at the end of the previous strands denoting *j*-job to *i*-individual at (4) step. And we let ||*w_ij_*|| = *c_ij_* *mer* and max ||*w_ij_*|| = *y mer*. Then *T*_2_ can be described:

$$sA_{i_{1}}eB_{j_{1}}sA_{i_{2}}eB_{j_{2}}\cdots sA_{i_{p}}eB_{j_{p}}\cdots sA_{i_{n}}eB_{j_{n}}w_{i_{1}j_{1}}w_{i_{2}j_{2}}\cdots w_{i_{p}j_{p}}\cdots w_{i_{n}j_{n}}$$

So the length range of DNA strands in tube *T*_2_ is:

$$\begin{array}{lll} \left\|S\right\| & = & \left\|s\right\|+\left\|A_{i_{1}}\right\|+\left\|e\right\|+\left\|B_{j_{1}}\right\|+\left\|s\right\|+\left\|A_{i_{2}}\right\|+\left\|e\right\|+\left\|B_{j_{2}}\right\|+\cdots \\ & & +\left\|s\right\|+\left\|A_{i_{n}}\right\|+\left\|e\right\|+\left\|B_{j_{n}}\right\|+\left\|w_{i_{1}j_{1}}\right\|+\left\|w_{i_{2}j_{2}}\right\|+\cdots \left\|w_{i_{n}j_{n}}\right\|\\ & = & \sum_{i=1}^{n}\left\|s\right\|+\sum_{k=1}^{n}\left\|A_{i_{k}}\right\|+\sum_{i=1}^{n}\left\|e\right\|+\sum_{k=1}^{n}\left\|B_{j_{k}}\right\|+\sum_{k=1}^{n}\left\|w_{i_{k}j_{k}}\right\|\\ & = & 4nt+\sum_{k=1}^{n}\left\|w_{i_{k}j_{k}}\right\| \end{array}$$

∵ 0 ≤ $w_{i_{k}j_{k}}$ ≤ *y*

∴ 4*tn* ≤ ||*T*_2_|| ≤ (4*t* + *y*)*n*

The length of strands in *T*_2_ tube must be between 4*tn* and (4*t* + *y*)*n*. So we can get the solution in step (4) in the appropriate length range.

## 3. Experimental Section

It is shown that errors in the separation of the library strands are errors in the computation [26,27,28,29,30,31,32]. This means that it needs a lower rate of primary errors of hybridization in the computation. DNA sequences should be generated to ensure library strands have little secondary structure that might inhibit intended probe-library hybridization. The design must also exclude DNA sequences that might encourage unintended probe-library hybridization. In order to realize the goals, we need to follow some DNA sequence constraints. For instance, library strands should include only A, T, and C, which has a lower secondary structure than those conditions and has higher chance to bind probes; through the restriction every library and probe sequence should have no runs of more than four A, four T, four C or four G, long homopolymer tracts which may have unusual secondary structures that inhibit the binding from probes to library strands and the melting temperatures of probe and library strand hybridization will be more similar, if they do not have any long enough homopolymer tracts. Restrictions that each probe should have four, five, or six G in its sequence are aimed to insure that intended probe-library pairings have uniform melting temperatures, and so on.

In this paper, the Adleman procedures [29] are simulated working on an electronic computer. The coded algorithms are used to generate DNA sequences to solve the unbalanced assignment problem and construct the 15-base DNA sequences for every bit of the library. For Table 1, the algorithm generates 5-base random sequences, consisting of *A_k_*, *B_k_*, *s*, *e*, and confirms whether the library strands satisfy above-mentioned restrictions when a new DNA sequence is gained [30]. If the restrictions are satisfied, the new DNA sequence is “greedily” taken up. If the restrictions are not satisfied, then mutations would be imported individually, in succession, into the new block until either the restrictions are satisfied and then the new DNA sequence is accepted, or a threshold for the number of mutations is reached, and the algorithm has finished and so it quits, printing the DNA sequence found so far. If the whole string of bits satisfy the restrictions, the algorithm has succeeded and these DNA sequences would be the product.

Consider the example in Table 1, the problem includes jobs {*j*_1_, *j*_2_, *j*_3_, *j*_4_, *j*_5_},and individuals {*i*_1_, *i*_2_, *i*_3_}. Basic DNA sequences are generated by Adleman’s algorithms and modified, shown in Table 8. Table 9 shows DNA sequences representing the job assignment schemes *sA_i_eB_j_* (1 ≤ *i* ≤ *m*, 1 ≤ *j* ≤ *n*). Adleman’s algorithms are also used to calculate the enthalpy, entropy, and free energy for binding of each probe to its corresponding region on a library strand; meanwhile, the energy used is shown in Table 10.

**Table 8:** Sequences chosen to represent *s*, *e*, *A_k_*, *B_k_*, and *w_ij_* in the example for Table 1.

Bit	3′–5′ DNA Sequence	Bit	3′–5′ DNA Sequence	Bit	3′–5′ DNA Sequence	Bit	3′–5′ DNA Sequence
*s*	CTATC	*e*	AACTC	*A*_1_	TAAAA	*B*_1_	AATTA
*A*_2_	CTTTT	*B*_2_	CATTA	*A*_3_	TTCAA	*B*_3_	ATCTA
*B*_4_	CAAAC	*B*_5_	ATCCA	*w*_11_	CCCAT	*w*_12_	ATATA
*w*_13_	TTACA	*w*_14_	TACCC	*w*_15_	TTCTT	*w*_21_	TTTCA
*w*_22_	ATAAT	*w*_23_	CTACC	*w*_24_	TTACA	*w*_25_	CATAC
*w*_31_	CCTTC	*w*_32_	ACTCA	*w*_33_	TCACT	*w*_34_	ACCCT
*w*_35_	TTAAC						

**Table 9:** Sequences chosen to represent the job assignment schemes *sA_i_eB_j_* (1 ≤ *i* ≤ *m*, 1 ≤ *j* ≤ *n*) in the example for Table 1.

Job Assignment	3′–5′ DNA Sequence	Job Assignment	3′–5′ DNA Sequence
*sA*_1_*eB*_1_	CTATCTAAAAAACTCAATTA	*sA*_1_*eB*_2_	CTATCTAAAAAACTCCATTA
sA_1_eB_3_	CTATCTAAAAAACTCATCTA	*sA*_1_*eB*_4_	CTATCTAAAAAACTCCAAAC
*sA*_1_*eB*_5_	CTATCTAAAAAACTCATCCA	*sA*_2_*eB*_1_	CTATCCTTTTAACTCAATTA
*sA*_2_*eB*_2_	CTATCCTTTTAACTCCATTA	*sA*_2_*eB*_3_	CTATCCTTTTAACTCATCTA
*sA*_2_*eB*_4_	CTATCCTTTTAACTCCAAAC	*sA*_2_*eB*_5_	CTATCCTTTTAACTCATCCA
*sA*_3_**eB**_1_	CTATCTTCAAAACTCAATTA	*sA*_3_**eB**_2_	CTATCTTCAAAACTCCATTA
*sA*_3_*eB*_3_	CTATCTTCAAAACTCATCTA	*sA*_3_*eB*_4_	CTATCTTCAAAACTCCAAAC
*sA*_3_*eB*_5_	CTATCTTCAAAACTCATCCA		

**Table 10:** The energies for of binding each probe to its corresponding region on a library strand.

Job Assignment	Enthalpy Energy *H*	Entropy Energy *S*	Free Energy *G*	Job Assignment	Enthalpy Energy *H*	Entropy Energy *S*	Free Energy *G*
*sA*_1_*eB*_1_	110.5	287.1	24.7	*sA*_1_*eB*_2_	101.2	256.7	22.9
*sA*_1_*eB*_3_	111.6	294.4	25.9	*sA*_1_*eB*_4_	107.4	281.3	24.9
*sA*_1_*eB*_5_	108.3	277.7	25.2	*sA*_2_*eB*_1_	104.2	271.3	24.9
*sA*_2_*eB*_2_	99.8	248.2	22.9	*sA*_2_*eB*_3_	105.4	270.3	24.8
*sA*_2_*eB*_4_	104.5	269.7	24.7	*sA*_2_*eB*_5_	97.7	243.1	23.3
*sA*_3_*eB*_1_	107.6	275.3	25.2	*sA*_3_*eB*_2_	111.2	289.5	25.8
*sA*_3_*eB*_3_	103.3	262.4	24.6	*sA*_3_*eB*_4_	114.7	292.1	26.3
*sA*_3_*eB*_5_	112.1	288.5	26.1				

Our program also figures out the average and standard deviation for the enthalpy, entropy, and free energy over all probe/library strand interactions. The energy levels are shown as in Table 11. Table 12 presents the library strands and the solution {*j*_3_→*i*_1_, *j*_4_→*i*_1_, *j*_2_→*i*_2_, *j*_1_→*i*_3_, *j*_5_→*i*_3_}of the unbalanced assignment problem.

**Table 11:** The energies over all probe/library strand interactions.

**Average**	106.463	273.613	24.794
**Standard Deviation**	4.8018	15.3631	1.0363

**Table 12:** DNA sequences chosen to represent the answer of the unbalanced assignment problem.

Solutions	DNA Strands Denoting Solutions
{*j*_3_→*i*_1_, *j*_4_→*i*_1_,*j*_2_→*i*_2_, *j*_1_→*i*_3_,*j*_5_→*i*_3_}	3′-CTATCTAAAAAACTCATCTACTATCTAAAAAACTCCAAAC CTATCCTTTTAACTCCATTACTATCTTCAAAACTCAATTACTAT CTTCAAAACTCATCCATTACATACCCATAATCCTTCTTAAC-5′

## 4. Conclusions

In this paper, we present DNA algorithms for solving the unbalanced assignment problem based on biological operations in the Adleman-Lipton model. Due to electronic computers having obvious limits in storage, speed, intelligence, and miniaturization, the methods of DNA computation have arisen, especially for their efficient parallelism. The present algorithm has the following advantages compared with previous algorithms: firstly, the proposed algorithm actually has a lower rate of errors for hybridization because we develop a computer program to generate good DNA sequences for generating the solution space of the unbalanced assignment problem. Secondly, the proposed algorithm requires a time cost and a DNA strand length that are linearly proportional to the instance size. It can finish in *O*(*mn*) the unbalanced assignment problem with n jobs and m individuals, having certain advantages comparing with existing algorithms, such as algorithm of paper [33] with *O*(*n*log*n*(*m* + *n*log*n*)) complexity. Additionally, the proposed algorithms can be easily performed in a fully automated manner in a laboratory. The full automation manner is essential not only for the speedup of computation but also for error-free computation. Meanwhile we simulated the DNA experiment to solve the unbalanced assignment problem. The ability to perform complex operations in solution might help us learn more about the nature of computation and lead to the development of better DNA-based computation, capable of solving a wide range of complex problems. We hope that, in future studies, more highly-effective DNA operations will be exploited to derive a DNA computing model with time efficiency and is complete for NP-hard problems.
